# Editorial: Rhizobiaceae mediated HGT: Facts, mechanisms, and evolutionary consequences

**DOI:** 10.3389/fpls.2023.1149426

**Published:** 2023-02-01

**Authors:** Tatiana Matveeva, Evgeny Аndronov, Ke Chen

**Affiliations:** ^1^ Department of Genetics and Biotechnology, Saint Petersburg State University, St Petersburg, Russia; ^2^ Microbial Monitoring Department, All-Russia Research Institute for Agricultural Microbiology, Podbelskogo sh. 3, Saint-Petersburg, Russia; ^3^ Shanghai Key Laboratory of Plant Functional Genomics and Resources, Shanghai Chenshan Botanical Garden, Shanghai, China

**Keywords:** horizontal gene transfer, *Rhizobium*, nitrogen fixing symbiosis, genospecies, population structure, *Agrobacterium*, T-DNA, naturally transgenic plants

Bacteria of the Rhizobiaceae family have been the focus of research for decades. One of the important reasons for this is the peculiarities of their interaction with plants, varying from parasitism (in the case of «*Agrobacterium*» species causing crown gall or hairy root diseases) to mutualism (in the case of *Rhizobium* species, causing the formation of nitrogen-fixing nodules). Studies of recent years indicate that the line between parasitism and mutualism in plant-rhizobial interactions is very arbitrary. Development of NGS and NNGS methods, accompanied by the emergence of new bioinformatics tools provides new opportunities for deeper study of genomes and transcriptomes of species, certain gene combinations and differentially expressed genes specific for the symbiotic interactions. These new data opened up new perspectives for studying of the evolutionary role of horizontal gene transfer for the formation and development of plant-rhizobial symbioses. Avalanche-like accumulating facts in favor of horizontal gene transfer involving rhizobia require systematization and new experimental approaches. that are reflected in this topic.

Opening a selection of Research Topic articles the review of Provorov et al. describes evolutionary aspects of nitrogen-fixing symbiosis. Authors propose the idea that microevolution of symbiotic nitrogen-fixing rhizobia is based on the diversification of *sym* genes occurring under the impacts of host-induced natural selection (including its disruptive, frequency-dependent and group forms). By contrast, macroevolution represents the polyphyletic origin of super-species taxa, which are dependent on the transfer of *sym* genes from rhizobia to various soil-borne bacteria., demonstrating evolution directed by the particular ecological environment of bacteria. *Rhizobia* in a different ecological environment are characterized by different determinants of the accessory genome. Thus, fungus-derived *Rhizobium*, described by Hua et al, lost almost all nitrogen fixation genes but specially acquired one redox cofactor cluster with *pqqE*, *pqqD*, *pqqC*, and *pqqB* involved in the synthesis of pyrroloquinoline quinone, participating in phosphate solubilization activity. The idea of the importance of the ecological environment for the formation of the accessory genome is also supported by the work of Wen et al.

Writing about the study of the mechanisms of horizontal gene transfer, it is widely accepted to attribute conjugation, transformation, and transduction to them. In recent years, methodological improvements have made it possible to assess the role of viruses in ecosystems at a new level, including their role as potential vectors for horizontal gene transfer. To achieve this goal, it is extremely important to study the biodiversity of phages, their host specificity and evidence of the alternation of lytic and lysogenic stages in the life cycle. An extensive study of single-stranded DNA phages of *Rhizobium* was performed by Cauwenberghe et al. Their phylogenetic analysis of the major capsid protein confirms the divergence of the *Rhizobium* microviruses into separate clades. At the same time the phylogeny of the bacterial hosts matches the microvirus phylogeny, suggesting a coevolution of phages and their bacterial hosts. The role of newly discovered phages in horizontal gene transfer between rhizobia may become the nearest prospect for their research.

Finally, representatives of the genus *Rhizobium* themselves can act as vector systems for horizontal transfer of their T-DNA and integration into plant genomes. Interest in this field has grown in recent years after describing the magnitude of the phenomenon of the emergence of full-fledged GMOs in nature without human intervention. In the article by Chen et al. an example of a group of related natural transgenic species of the *Camellia* genus is considered. In this research work, the allelic assembly of natural transgenes was carried out for the first time. This made it possible to use the data obtained to clarify the phylogeny of the *Thea* section of *Camellia*. The study of clustering of allelic variants on the phylogenetic tree made it possible to distinguish two groups of sequences that accumulated mutations leading to the loss of genes function from a common intact ancestral sequence. Such approaches may make it possible to further understanding under what conditions natural transgenes could function, and under what changes in the ecological environment the loss of gene functions occurred.

One of the important thoughts that can generalize the results obtained will be, that both parasitic and mutualistic interactions between representatives of different groups of living organisms (pro- and eukaryotes, viruses) involve very close cohabitation, and, as it turns out, one of the essential aspects of this co-existence is not only the physiological and biochemical integration of partners, but also their evolutionary and genetic integration. The latter implies a certain degree of creating of common DNA pool, one of the mechanisms of which is horizontal gene transfer. The evolutionary meaning of horizontal gene transfer, we believe, will be the subject of intensive research for many years to come.

## Author contributions

All authors listed have made a substantial, direct, and intellectual contribution to the work and approved it for publication.

